# Clinical characteristics and molecular genetic analysis of 22 patients with neonatal diabetes from the South-Eastern region of Turkey: predominance of non-K_ATP_ channel mutations

**DOI:** 10.1530/EJE-14-0852

**Published:** 2015-06

**Authors:** Huseyin Demirbilek, Ved Bhushan Arya, Mehmet Nuri Ozbek, Jayne A L Houghton, Riza Taner Baran, Melek Akar, Selahattin Tekes, Heybet Tuzun, Deborah J Mackay, Sarah E Flanagan, Andrew T Hattersley, Sian Ellard, Khalid Hussain

**Affiliations:** 1 Departments of Paediatric Endocrinology, Great Ormond Street Hospital for Children NHS Trust, London, WC1N 3JH, UK; 2 The Institute of Child Health, University College London, London, WC1N 1EH, UK; 3 Departments of Paediatric Endocrinology, Children State Hospital, Diyarbakir, 21100, Turkey; 4 Institute of Biomedical and Clinical Science, University of Exeter Medical School, Exeter, EX2 5DW, UK; 5 Departments of Neonatology, Children State Hospital, Diyarbakir, 21100, Turkey; 6 Department of Medical Biology and Genetics, Dicle University, Diyarbakir, 21100, Turkey; 7 Faculty of Medicine, University of Southampton, Southampton, SO16 6YD, UK

## Abstract

**Background:**

Neonatal diabetes mellitus (NDM) is a rare form of monogenic diabetes and usually presents in the first 6 months of life. We aimed to describe the clinical characteristics and molecular genetics of a large Turkish cohort of NDM patients from a single centre and estimate an annual incidence rate of NDM in South-Eastern Anatolian region of Turkey.

**Design and methods:**

NDM patients presenting to Diyarbakir Children State Hospital between 2010 and 2013, and patients under follow-up with presumed type 1 diabetes mellitus, with onset before 6 months of age were recruited. Molecular genetic analysis was performed.

**Results:**

Twenty-two patients (59% males) were diagnosed with NDM (TNDM-5; PNDM-17). Molecular genetic analysis identified a mutation in 20 (95%) patients who had undergone a mutation analysis. In transient neonatal diabetes (TNDM) patients, the genetic cause included chromosome 6q24 abnormalities (*n*=3), *ABCC8* (*n*=1) and homozygous *INS* (*n*=1). In permanent neonatal diabetes (PNDM) patients, homozygous *GCK* (*n*=6), *EIF2AK3* (*n*=3), *PTF1A* (*n*=3), and *INS* (*n*=1) and heterozygous *KCNJ11* (*n*=2) mutations were identified. Pancreatic exocrine dysfunction was observed in patients with mutations in the distal *PTF1A* enhancer. Both patients with a *KCNJ11* mutation responded to oral sulphonylurea. A variable phenotype was associated with the homozygous c.-331C>A *INS* mutation, which was identified in both a PNDM and TNDM patient. The annual incidence of PNDM in South-East Anatolian region of Turkey was one in 48 000 live births.

**Conclusions:**

Homozygous mutations in *GCK*, *EIF2AK3* and the distal enhancer region of *PTF1A* were the commonest causes of NDM in our cohort. The high rate of detection of a mutation likely reflects the contribution of new genetic techniques (targeted next-generation sequencing) and increased consanguinity within our cohort.

## Introduction

Neonatal diabetes mellitus (NDM) is a rare form of monogenic diabetes which presents in the first 6 months of life (1). There are two main clinical subtypes of NDM: permanent neonatal diabetes (PNDM) and the remitting and frequently relapsing, transient neonatal diabetes (TNDM) (2, 3). The underlying genetic defect in TNDM can be ascertained in more than 90% cases. The majority of cases of TNDM are linked to an imprinted region on chromosome 6q24, the abnormality either being paternal uniparental disomy, paternal duplication or defective methylation of the maternal allele (4). The latter can sometimes be due to biallelic mutations in *ZFP57*, a gene involved in the regulation of DNA methylation (5). Heterozygous mutations in genes encoding the KIR6.2 and SUR1 subunits of the pancreatic ATP-sensitive potassium (K_ATP_) channels (*KCNJ11* and *ABCC8*) are common causes of TNDM (6).

PNDM is genetically heterogeneous with mutations in 20 different genes described to date: *KCNJ11*, *ABCC8*, *FOXP3*, *GCK*, *PDX1*, *PTF1A*, *EIF2AK3*, *SLC2A2*, *GATA6*, *SLC19A2*, *WFS1*, *NEUROD1*, *NEUROG3*, *RFX6*, *WFS1*, *NKX2-2*, *MNX1*, *IER3IP1*, *INS* and *GL* *IS3* (1, 7). Heterozygous mutations in the *KCNJ11*, *ABCC8* and *INS* gene are the most common causes of PNDM. However, in populations with a high degree of consanguinity, rare genetic causes of PNDM such as homozygous mutations in *EIF2AK3*, *INS* and *GCK* account for the majority of cases (8).

The incidence of NDM in different countries has been calculated from the referral rate of new cases to the diagnostic laboratory or referral endocrine centres. Thus reported incidences are quite variable (one in 21 196 to one in 215 417) not only depending on the calculation method but also the population characteristics (e.g. prevalence of consanguinity) (9, 10, 11). In general, the incidence in western countries is lower than the eastern countries with a high prevalence of consanguinity. Turkey, particularly South-Eastern Anatolian region, is an area with a high prevalence (>40%) of consanguineous marriage (12).

To the best of our knowledge, the regional and nationwide annual incidence of NDM has not been reported from Turkey. We describe the clinical characteristics, molecular genetics and long-term follow-up of a large Turkish cohort of NDM patients from a single paediatric endocrine centre. We also estimate the incidence of NDM in South-Eastern Anatolian region of Turkey based on referrals to a regional paediatric endocrine centre.

## Subjects and methods

### Patients

Patients referred/presenting with NDM to the paediatric endocrine department at Diyarbakir Children State Hospital, Turkey between January 2010 and December 2013 were phenotyped in detail. The paediatric endocrinology department at Diyarbakir Children State Hospital is the only paediatric endocrine centre in Diyarbakir and also receives NDM referrals from four neighbouring cities (Siirt, Sirnak, Mardin and Batman) in the South-Eastern Anatolian region.

NDM was defined as onset of diabetes mellitus diagnosed before 6 months of age. Details of clinical presentation, birth and family history, clinical phenotype, biochemical data, pancreatic imaging and management were collected using a standardised proforma.

Molecular genetic analysis was undertaken as described below. In addition, genetic analysis was undertaken on patients with presumed type 1 diabetes mellitus under follow-up where the clinical phenotype was consistent with monogenic diabetes.

### Incidence of NDM

The annual incidence of NDM was calculated for the South-Eastern Anatolian region of Turkey between 2010 and 2013 inclusive, based on referral rates of NDM to the tertiary paediatric endocrine centre in Diyarbakir. The annual live birth rate data for this region was supplied by Turkish Statistical Institute (TSI; http://tuikapp.tuik.gov.tr/demografiapp/dogum.zul).

### Genetic analysis

Genomic DNA was extracted from peripheral leukocytes of 19 patients using standard procedures, and the coding regions and intron/exon boundaries of the *ABCC8*, *KCNJ11*, *INS* and *EIF2AK3* genes were amplified by PCR (primers available on request). The amplicons were sequenced using the Big Dye Terminator Cycler Sequencing Kit v3.1 (Applied Biosystems) according to manufacturer's instructions, and reactions were analysed on an ABI 3730 Capillary sequencer (Applied Biosystems). The sequences were compared with the reference sequences (NM_000525.3, NM_000352.3 (U63421 and L78208), NM_000207.2 and AF110146.1) using Mutation Surveyor v3.24 Software (SoftGenetics, State College, PA, USA).

For one patient, Sanger sequencing of the coding regions of *GCK* (NM_000162.3) was undertaken. This was prompted by the presence of mild-fasting hyperglycaemia in both parents. In a second patient with pancreatic exocrine insufficiency, sequence analysis of a distal *PTF1A* regulatory enhancer region was performed by methods described previously (13).

All 20 known neonatal diabetes genes were subsequently screened on the Illumina HiSeq2000 targeted next-generation sequencing platform in six patients where Sanger sequencing, or methylation analysis, had not identified a causative mutation. Details of the methodology have been reported previously (7).

Chromosome 6q24 methylation analysis was performed on three patients with TNDM, in whom *ABCC8*, *KCNJ11*, *INS* and *EIF2AK3* mutations had been excluded. Methylation-specific PCR was used to detect hypomethylation of the 6q24 locus, followed by microsatellite analysis to discriminate uniparental disomy of chromosome 6 (UPD6pat) from isolated hypomethylation at 6q24, as described (14). The samples with 6q24 hypomethylation but not UPD6pat were tested for hypomethylation at other imprinted loci and for *ZFP57* mutations, as described (5).

The study was performed according to the principles of the Declaration of Helsinki with written informed consent given by the patients' parents.

### Statistical analyses

Statistical analysis was performed using IBM SPSS 21.0 for Windows statistical software. Shappiro–Wilk test was used to test the normality of distribution of data. The ratios were compared using *χ* ^2^ test (or Fisher's exact test). The means were compared using ‘independent sample *t*-test’ in normally distributed data and medians using Mann–Whitney *U* test for non-normally distributed data. Data were expressed as median (interquartile range) or mean±s.d. (range). A *P* value ≤0.05 was considered to be statistically significant.

## Results

### Presenting characteristics

Twenty-two patients were diagnosed with NDM. Seventeen patients were born between January 2010 and December 2013. Five patients were born before January 2010 but were not tested for mutations at the time of diagnosis of NDM. Three of them had been misdiagnosed as type 1 diabetes mellitus, whereas two died in early infancy.

NDM resolved in five patients (TNDM), whereas 15 patients had PNDM. Two patients died in early infancy due to unrelated illnesses and hence could not be clearly classified as TNDM or PNDM. However, due to a strong indication of GCK-NDM, we classified these patients into the PNDM group. Comparison of the two subtypes revealed significant difference in the age at presentation of diabetes, with TNDM patients presenting earlier than PNDM patients (Table 1). Incidentally, all TNDM patients in our cohort were females, whereas for all types of NDM, the male-to-female ratio was 13:9.

Patients with *EIF2AK3* and K_ATP_ channel mutations were appropriate for gestational age (birth weight between −2 s.d. and +2 s.d.), whereas patients with mutations in *GCK*, *PTF1A*, *INS* and 6q24 methylation abnormality were small for gestational age (birth weight <−2 s.d.) (Table 2).

### Annual incidence of NDM

According to TSI, the total number of live births in the five cities (Diyarbakir, Siirt, Sirnak, Mardin and Batman) in the South-Eastern Anatolian region of Turkey between 2010 and 2013 was 387 857. In our cohort of 22 NDM patients, 13 patients (five TNDM and eight PNDM) were born during this period across these five cities. These figures suggest that the overall annual incidence of NDM (and PNDM) during this period in the South-Eastern Anatolian region of Turkey was at least one in 30 000 (one in 48 000) live births.

### Mutation analysis

**Figure 1:**
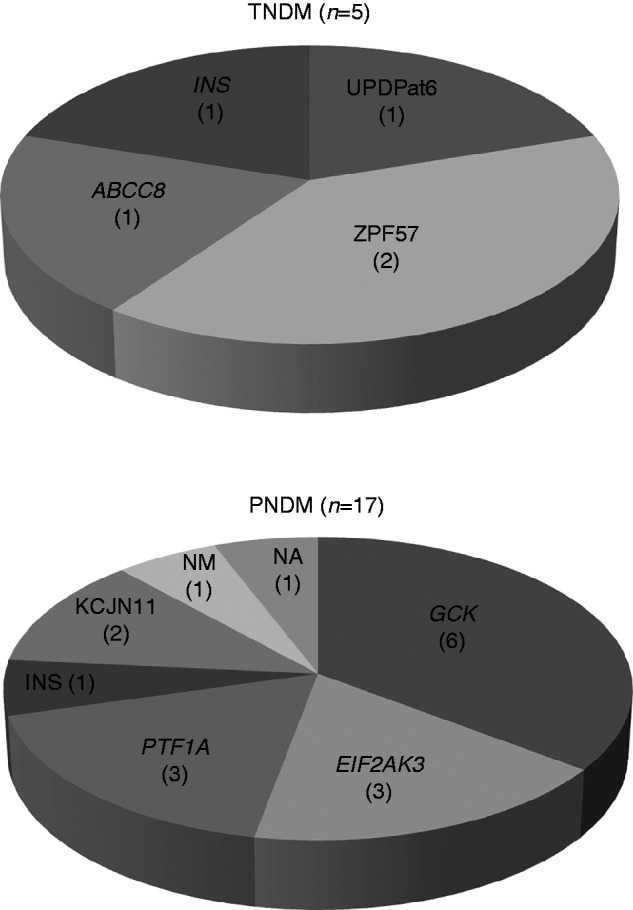
Distribution of mutations detected in patients with TNDM and PNDM (NA, not available; NM, no mutation).

The underlying genetic cause for NDM was identified in 20/21 (95%) patients by undergoing a mutation anlysis. Chromosome 6q24 abnormalities, heterozygous *ABCC8* and homozygous *INS* mutations were identified in three, one and one patients respectively (Fig. 1). Two patients with chromosome 6q24 methylation abnormalities had a homozygous mutation in *ZFP57* (p.Q322RfsX13 and p.R228C) and one patient had paternal uniparental isodisomy (UPD) of chromosome 6. The p.Q322RfsX13 (c.964delC) frameshift mutation is a single-base deletion which results in the introduction of a premature termination codon. The p.R228C (c.682C>T) mutation is a missense mutation which is predicted to change the conformation of a *cys*-*his* zinc finger 3.

The genetic cause of diabetes was identified in 15 of the 17 PNDM patients. Nine different mutations in five genes (*GCK*, *EIF2AK3*, *PTF1A*, *KCNJ11* and *INS*) were identified (Fig. 1). Two different homozygous *GCK* mutations (p.K169R (c.506A>G) and p.C220R (c.658T>C)) were identified in four patients, two of whom were siblings. A further two NDM patients who died in early infancy of unrelated illnesses, siblings of patient 2, are likely to have had *GCK* NDM (although not formally tested) as both parents were heterozygous for the p.K169R *GCK* mutation. Both mutations were novel at the time of diagnosis, and affect residues which are highly conserved across species. A different mutation at the same amino acid residue has been reported previously (15).

Three different homozygous *EIF2AK3* mutations were identified in three patients, one of which, p.W521X (c.1562G>A), has been reported previously (8, 16). The remaining two mutations were novel (c.1884-1G>C and p.Q333X (c.997C>T)).

Two different homozygous mutations (chr10:g.23508437 and chr10:g.23508365) in the distal enhancer region of *PTF1A* (∼25 kb downstream from *PTF1A*) were identified in three patients. Both mutations have been reported previously in patients with pancreatic hypoplasia (13).

A previously reported heterozygous *KCNJ11* mutation (p.R201H, c.602G>A) was identified in two unrelated patients (17). One patient had a previously reported homozygous *INS* promoter mutation (c.-331C>A) which is predicted to disrupt a transcriptional regulatory site resulting in decreased insulin transcription (18).

No mutation was identified in two patients, one patient had been tested by targeted next-generation sequencing (7). There was inadequate DNA available for testing for the remaining patient.

### Clinical details and genotype–phenotype relation

All six patients with a homozygous *GCK* mutation were born to consanguineous families and the median age of presentation with diabetes was 5 days (range 2–28 days). One patient had been misdiagnosed as type 1 diabetes mellitus and confirmation of *GCK* NDM was done at a later age rather than at initial presentation. Two siblings of this patient were diagnosed with NDM, but died in early infancy. It is presumed that they also had the identical homozygous *GCK* mutation. All these patients are currently on a relatively high dose of s.c. insulin (1.5 U/kg per day). In a trial to switch s.c. insulin therapy to sulphonylurea, none of these patients responded. Coincidentally, one patient also had thalassemia major and required regular blood transfusions.

The three patients with Wolcott–Rallison syndrome (WRS), due to homozygous *EIF2AK3* mutations, were from consanguineous families and they presented with diabetes at a median age of 85 days (range 66–96 days). Over a mean follow-up period of 2.5 years, episodic elevation of liver enzymes and skeletal abnormalities were noticed in two patients. Imaging revealed pancreatic hypoplasia in one patient who died at the age of 3 years due to acute liver failure (Table 2).

The three patients with mutations in distal enhancer region of *PTF1A* presented with diabetes at a median age of 3 weeks (range 1–10 weeks). In addition to NDM, all three patients displayed symptoms of pancreatic exocrine insufficiency and pancreatic hypoplasia on imaging.

The two patients with *KCNJ11* mutations presented with diabetes between 1 and 3 months of age. One of these patients had additional neurological features including epilepsy and developmental delay, which developed after acute presentation with diabetic ketoacidosis. This patient was misdiagnosed as type 1 diabetes mellitus and the confirmation of *KCNJ11* NDM was done at the age of 6.2 years. An attempt was made to switch both patients to an oral sulphonylurea therapy after the molecular genetics diagnosis. Both patients responded well to glibenclamide (0.5 and 1.5 mg/kg per day respectively) and insulin therapy was successfully weaned.

The patient with an *ABCC8* mutation presented at 18 days of age and the diabetes remitted at the age of 7 months. She is now 2.4 years of age and has a normal blood glucose profile. Post remission, her HbA1c improved from 7.1% (54 mmol/mol) to 5.2% (33 mmol/mol).

Both patients with the homozygous *INS* promoter mutation presented with NDM within the first week of life. The mean birth weight was 1650 g at 36 weeks gestational age. One patient remitted at the age of 65 days, whereas the second patient, 13 months at the time of writing, was on insulin therapy.

All three patients with NDM due to chromosome 6q methylation abnormalities presented within first week of life. The mean birth weight was 2440 g at 40 weeks gestational age. All three patients remitted before 5 months of age. One patient with TNDM due to paternal UPD died due to unrelated illness. The remaining two patients with a *ZFP57* mutation, aged 1 and 3 years, are still in remission. Apart from macroglossia in one patient, no other additional features were observed.

For the two patients in whom the underlying genetic cause could not be established, there is a strong family history of diabetes mellitus in at least three generations for one patient (proband, father, brother and grandfather). None of the other diabetic members from his family had been diagnosed during the neonatal period. Mutation analysis of all 20 known NDM genes did not identify a mutation in this patient. In the second patient, who died at the age of 2.5 years, apart from NDM there were additional features of thiamine-responsive megaloblastic anaemia, pancreatic exocrine insufficiency and neurodevelopmental delay. No improvement in insulin requirement was noticed with thiamine replacement. DNA was not available from this patient.

## Discussion

We describe the genetic aetiology and clinical manifestations of NDM patients from a single, large paediatric endocrine centre from South-Eastern Anatolian region of Turkey. To the best of our knowledge, our centre's cohort of 22 NDM patients is the largest single centre cohort, 20 of whom have received a genetic diagnosis.

In our study, mutations in *GCK*, *EIF2AK3* and a distal enhancer region of *PTF1A* were the commonest cause of NDM, accounting for 30, 15 and 15% of genetically confirmed cases respectively. Although homozygous inactivating mutations in *GCK* are a rare cause of PNDM, isolated cases of *GCK* PNDM are frequently reported from consanguineous pedigrees (19, 20, 21). However, a recent study from Saudi Arabia, where there is a high degree of consanguinity, did not identify a single case of *GCK* PNDM (22). Another study looked at the genetic aetiology of Arab and British cohorts tested in Exeter Peninsula Medical School Genetics Laboratory between years 2006 and 2011 and found *GCK* PNDM in only five out of 88 Arabic patients (5.7%) and one out of 77 British patients (1.3%) tested (23). To the best of our knowledge, there have not been any previous large case-series from Turkey reporting the genetic cause of NDM. From our results, it seems that *GCK* PNDM is the commonest cause of NDM in South-Eastern Anatolian region of Turkey and accounts for more than one-quarter of NDM cases. This is the highest proportion of *GCK* PNDM reported to date. Our patients with homozygous *GCK* mutations did not respond to the trial of sulphonylurea therapy. We also noticed higher requirement of insulin (1.5–2 U/kg per day) to maintain normoglycaemia in *GCK* PNDM as compared with other NDM patients.

The incidence of TNDM caused by mutation of *ZFP57* was also high in our cohort. A recent study of a global cohort (*n*=163) of TNDM reported 12 patients (7.5%) with mutation in *ZFP57* (24), of which ten had homozygous mutations from consanguineous pedigrees and only two had heterozygous mutations derived from unrelated parents (Mackay 2014 unpublished data). In the cohort presented here, two out of the three TNDM cases with chromosome 6q24 methylation abnormalities, had *ZFP57* mutations. It is apparent that the prevalence of *ZFP57* mutations correlated with that of consanguinity; this should be borne in mind when TNDM is suspected because the recurrence risk in affected pedigrees is 25%.

WRS (*EIF2AK3* mutations) has been reported as the most common cause of PNDM in consanguineous families (8). Pancreatic agenesis/hypoplasia, acute episodes of liver dysfunction and skeletal dysplasia during follow-up are major clinical characteristics of WRS that have significant influence on prognosis (8, 16, 24). Episodes of liver dysfunction, ranging from mild elevation of serum transaminases to severe hepatic failure, can develop at any time from diagnosis (8, 16, 24). In our series with a high percentage of consanguineous families, WRS was the second most common cause after *G* *CK* PNDM. In our cohort, one patient with a normal pancreas on ultrasound imaging developed a self-limiting mild elevation of transaminases, alanine transaminase and aspartate transaminase at presentation. The second patient with normal pancreatic magnetic resonance imaging did not develop any liver dysfunction. However, a skeletal dysplasia was noticed at the age of 1.5 year, which worsened during follow-up. The third patient had pancreatic hypoplasia on ultrasound imaging, developed hepatic failure and skeletal dysplasia at the age of 1 year and died at the age of 3 years due to the second episode of hepatic failure.

In our series, three patients from consanguineous pedigrees with clinical manifestations of PNDM and pancreatic exocrine insufficiency and pancreatic hypoplasia/agenesis were found to have mutations in the recently identified distal enhancer region of *PTF1A* (13). Our results suggest that mutations in this regulatory region of *PTF1A* are a relatively common cause of PNDM in consanguineous families. No additional clinical features were present in these patients, consistent with the reported clinical phenotype of isolated pancreatic agenesis with distal enhancer *PTF1A* mutations (13).

Although mutations in the *KCNJ11* and *ABCC8* genes are the commonest cause of PNDM in European and Japanese populations, in our study they accounted for 12.5% cases only (9, 25, 26, 27). Similar findings were reported in the case-series from Saudi Arabia with 17 PNDM patients, in whom no *KCNJ11* or *ABCC8* mutations were identified (22). Both of our patients carrying the p.R201H *KCNJ11* mutation could be switched to oral sulphonylurea with distinctly different doses of glibenclamide. It is well recognised that not all *KCNJ11* mutations respond to oral sulphonylurea therapy (28). Although it seemed that one patient had DEND phenotype, on careful assessment developmental delay and epilepsy in this particular patient are likely to be due to neurological insult sustained during acute prolonged period of diabetic ketoacidosis coma. No cases of DEND have been reported in association with p.R201H *KCNJ11* mutation in the literature.

Our results suggest that the estimated incidence of NDM in South-Eastern Anatolian region of Turkey was higher than those of western European countries with low rates of consanguineous marriage, but similar to the incidence reported from the Arabic countries with high rates of consanguineous marriage (9, 10, 11, 22, 29). The high rate of consanguinity in South-Eastern Anatolian region of Turkey and in our cohort (89%) was consistent with these results (12). However, since South-Eastern Anatolian region has a higher rate of consanguineous marriage compared with the other regions of Turkey, this estimated incidence may not reflect the true incidence of NDM for the whole Turkish population (12). To determine the true incidence rate of NDM in Turkey, multicentre and larger nationwide studies are required.

## Conclusions

We present the largest cohort of NDM from a single paediatric endocrine centre. Mutations in *GCK*, *EIF2A* *K3* and the distal enhancer region of *PTF1A* were the commonest causes of NDM in our cohort. With the utilisation of candidate gene sequencing and targeted next-generation sequencing, the underlying genetic cause could be established in 95% of our NDM patients who had undergone a mutation analysis. The high incidence and predominance of non-KATP channel mutations are likely to reflect the increased rate of consanguinity within our cohort. Special care to the rare genetic causes in the molecular genetic analysis of NDM is clearly required in patients from consanguineous pedigrees.

## Figures and Tables

**Table 1:** Comparison of clinical characteristics for patients with TNDM and PNDM.

	**TNDM** (*n*=5)	**PNDM** (*n*=17)	***P***
Age at presentation (weeks)	1.4±0.9	6.2±5.5	0.005
Gestational age (weeks)	38.8±2.2	38.2±2.7	0.499
Birth weight (g)	2388±526	2213±626	0.596
Plasma glucose (mmol/l)	31.1±9.4	28.3±10.6	0.884
C peptide (pmol/l)	0.7±0.8	0.3±0.3	0.690
Serum insulin (mU/l)	5.2±1.5	4.2±3.2	0.345
Female/male	5/0	4/13	0.006
DKA at presentation (*n*, %)	1/4 (25)	2/5 (40)	0.166
Monogenic diabetes in FH (*n*, %)	2/5 (40)	10/14 (71)	0.126
Consanguinity in FH, *n* (%)	5/5 (100)	12/14 (86)	0.544

DKA, diabetic ketoacidosis; FH, family history.

**Table 2:** Genotype–phenotype analysis and follow-up characteristics of mutation positive NDM patients.

**Family and patient no.**	**Gene**	**Age** ^a^ (weeks)	**GW/BW** (weeks/g)	**Current age** (years)	**Site of mutation**	**DNA** (protein) description	**Zygosity/novelty**	**NDM subtype**	**F/H MD**	**EPI**	**Pancreas imaging**	**SU-R**	**Associated disease and follow-up**
1.1	*GCK*	4	38/1600	2.4	Exon 5	c.506A>G (p.K169R)	HM/novel	Permanent	Yes	No	Normal	No	Thalassemia major
2.2	*GCK*	1	40/1900	6.3	Exon 5	c.506A>G (p.K169R)	HM	Permanent	Yes	No	Normal	No	Two siblings (patient 2.3 and 2.4) also had NDM
2.3	*GCK*	2	40/1700	Died	Exon 5	c.506A>G (p.K169R)^b^	HM	Permanent^b^	Yes	NA	Normal	NA	Died due to sepsis at 3 months
2.4	*GCK*	4	39/1600	Died	Exon 5	c.506A>G (p.K169R)^b^	HM	Permanent^b^	Yes	NA	Normal	NA	Died due to intestinal obstruction (post surgery) at 3 months
3.5	*GCK*	2	40/2400	2.7	Exon 6	c.658T>C (p.C220R)	HM/novel	Permanent	Yes	No	Normal	No	Patient 3.5 and 3.6 are siblings in a large consanguineous family with a number of individuals with monogenic diabetes
3.6	*GCK*	1	36/1600	1.2	Exon 6	c.658T>C (p.C220R)	HM	Permanent	Yes	No	Normal	No
4.7	6q24 (*ZFP57*)	1	40/3150	3.0	Exon 6	c.682C>T (p.R228C)	HM/novel	Transient	Yes	No	Normal	NA	Remission at 3 months
5.8	6q24 (*ZFP57*)	1	40/2200	1.0	Exon 6	c.964delC (p.Q322RfsX13)	HM/novel	Transient	No	No	Normal	NA	Macroglossia, remission at 5 months
6.9	6q24	1	39/1980	Died		Complete loss of methylation	UPDPat6	Transient	No	No	Normal	NA	Remission at 3 months, died when she was 5 months
7.10	*PTF1A*	1	31/1500	3.1	Promoter	g.23508437A>G	HM	Permanent	Yes	Yes	Agenesis	NA	Developmental delay
8.11	*PTF1A*	10	39/2400	3.1	Promoter	g.23508437A>G	HM	Permanent	No	Yes	Agenesis	NA	
9.12	*PTF1A*	3	32/1200	2.4	Promoter	g.23508365A>G	HM	Permanent	No	Yes	Agenesis	NA	Neonatal cholestasis
10.13	*EIF2AK* *3*	14	40/3000	Died	Exon 5	c.997C>T (p.Q333X)	HM	Permanent	Yes	No	Hypoplasia	NA	A hepatic failure observed at 12 months old, skeletal dysplasia on X-rays, died at the age of 3 years due to second attack of hepatic failure
11.14	*EIF2AK3*	12	40/2800	3.3	Exon 9	c.1562G>A (p.W521X)	HM/novel	Permanent	No	No	Normal	NA	No liver dysfunction observed, severe skeletal dysplasia
12.15	*EIF2AK3*	10	40/3050	1.0	Intron 11	c.1884-1G>C (p.?)	HM/novel	Permanent	Yes	No	Normal	NA	Transient elevation of liver enzymes, hypoalbuminaemia, no skeletal dysplasia at the 1 year of age
13.16	*INS*	1	35/1910	1.2	Promoter	c.-331C>A (p.?)	HM	Transient	No	No	Normal	NA	Remission at the age of 2 months
14.17	*INS*	1	37/1400	0.7	Promoter	c.-331C>A (p.?)	HM	Permanent	Yes	No	Normal	NA	No remission
15.18	*KCNJ11*	2	39/3000	4.8	Exon 1	c.602G>A (p.R201H)	HT	Permanent	Yes	No	Normal	Yes	Successful transfer to SU therapy and weaned off insulin therapy
16.19	*KCNJ11*	13	40/2800	6.6	Exon 1	c.602G>A (p.R201H)	HT	Permanent	No	No	Normal	Yes	Developmental delay, epilepsy, successful transfer to SU therapy and weaned off insulin therapy
17.20	*ABCC8*	3	40/2700	2.4	Exon 10	c.1594A>G (p.S532G)	HT/novel	Transient	Yes	No	Normal	NA	Remission at the age of 3 months

GW, gestation week; BW, birth weight; NDM, neonatal diabetes mellitus; HM, homozygous; HT, heterozygous; UPDPat6, paternal uniparental disomy on Chr6q24; F/H MD, family history of monogenic diabetes; EPI, exocrine pancreas insufficiency; SU-R, sulphonylurea response; SU, sulphonylurea.

a Age at NDM diagnosis.

b Not tested but presumably *GCK* PNDM as sibling diagnosed with *GCK* PNDM and both parents were heterozygous carriers of *GCK* mutation.
